# Editorial: Cancer Nanotheranostics: What Have We Learned So Far?

**DOI:** 10.3389/fchem.2015.00071

**Published:** 2016-01-06

**Authors:** João Conde, Furong Tian, Jesus M. de la Fuente, Pedro V. Baptista

**Affiliations:** ^1^Harvard-MIT Division for Health Sciences and Technology, Institute for Medical Engineering and Science, Massachusetts Institute of TechnologyCambridge, MA, USA; ^2^School of Engineering and Materials Science, Queen Mary University of LondonLondon, UK; ^3^School of Food Science and Environmental Health, College of Sciences and Health, Dublin Institute of TechnologyDublin, Ireland; ^4^Instituto de Ciencia de Materiales de Aragón, Consejo Superior de Investigaciones Científicas/Universidad de ZaragozaZaragoza, Spain; ^5^Research Unit on Applied Molecular Biosciences (UCIBIO), Departamento de Ciências da Vida, Faculdade de Ciências e Tecnologia, Universidade Nova de LisboaLisboa, Portugal

**Keywords:** oncology, cancer, nanotechnology, nanotheranostics, nanomaterials, nanoparticles

According to the *National Cancer Institute*, in 2015 an estimated of 1.7 million new cases of cancer will be diagnosed only in the United States and around 600,000 people will die from the disease. The most common type of cancer is breast cancer, with more than 234,000 new cases expected in the United States in 2015. The next most common cancers are prostate cancer and lung cancer.

After a quarter of century of rapid technological advances, research has revealed the complexity of cancer, a disease intimately related to the dynamic transformation of the genome. These transformations trigger a range of modification to cell processes and molecular events that initiate and promote tumor genesis and progression, then local invasion and metastasis, i.e., the hallmarks of cancer development. These alterations may cause a wide scope of “diseases” that share similar molecular patterns that cause transformation and malignancy. Each of this stepwise evolution of the initial molecular event drives abnormal growth and loss of differentiation that ultimately causes tissue and organ failure. The initial molecular event may lay within the erroneous expression of a given gene, epigenetic modification and/or sporadic mutations occurring on genomic DNA during the life span of organisms. Each and every one of these molecular events may be evaluated and used as diagnostics biomarker and therapeutic target. For example, therapy action may target a mutated gene and silence its expression so as to avoid erroneous protein expression that mutates cell function. However, the full understanding of the molecular onset of this disease is still far from achieved and the search for mechanisms of treatment will follow closely.

It is here that Nanotechnology enters the fray offering a wealth of tools to diagnose and treat cancer. Today, Nanotechnology is a burgeoning field that is helping to address critical global problems from cancer treatment to climate change. In fact, Nanotechnology is everywhere and is everyday practice (Conde), offering numerous tools to diagnose and treat cancer, such as new imaging agents, multifunctional devices capable of overcome biological barriers to deliver therapeutic agents directly to cells and tissues involved in cancer growth and metastasis, and devices capable of predicting molecular changes to prevent action against precancerous cells (Conde et al., 2012). The novel physical properties of inorganic particles at the nanometer size scale, combined with the high specific surface of polymeric nanoparticles and the possibility to engineer stimuli-respond drug release strategies, have provided new tools to physicians for the diagnostic and the therapy of diseases such as cancer.

Nanomaterials-based delivery systems in Theranostics (Diagnostics and Therapy), at the same size level of proteins, DNA or RNA, provide better penetration of therapeutic and diagnostic substances within the body at a reduced risk in comparison to conventional therapies. At the present time, there is a growing need to enhance the capability of theranostics procedures where innovative multifunctional nanocarriers for cancer theranostics may allow the development of diagnostics systems such as colorimetric and immunoassays, and in therapy approaches through gene therapy, drug delivery and tumor targeting systems in cancer (Conde et al., 2014).

Some of the thousands and thousands of published nanosystems so far will most likely revolutionize our understanding of biological mechanisms and push forward the clinical practice through their integration in future diagnostics platforms. Nevertheless, despite the significant efforts toward the use of nanomaterials in biologically relevant research, more *in vivo* studies are needed to assess the applicability of these materials as delivery agents. In fact, only a few have gone through feasible clinical trials. Nanomaterials have to serve as the norm rather than an exception in the future conventional cancer treatments. Future *in vivo* work will need to carefully consider the correct choice of chemical modifications to incorporate into the multifunctional nanocarriers to avoid activation off-target, side effects and toxicity.

It is imperious to learn how advances in nanosystems' capabilities are being used to identify new diagnostic and therapy apparatuses driving the development of personalized and precision medicine in cancer therapy and diagnostics; learn how incorporating cancer research and nanotechnology can help patient life quality; identify how to decipher nanotheranostics data into a real clinical strategy; and, last but not least, learn what methods are showing fertile results in turning promising clinical data into treatment realities (Conde et al., 2014).

Although all studies described in this Topic provide a baseline level of data in support of the effectiveness and safety of nanomaterials, we wonder what have we learned so far?

Current trends in biomedicine have been focused toward the use of new materials capable to address particular and individual characteristics in strategies for molecular precision therapies. In this endeavor, nanoparticles have allowed a tremendous leap forward in combining diagnostics and therapy in a single system and doing so at the nanoscale. Nanotheranostics have enabled the integration of targeting, imaging and therapeutics in a single platform, with proven applicability on the management of heterogeneous diseases.

Despite the plethora of proposed systems, only but a few products are currently included clinical trials and much remains to be done to allow effective clinical translation of these promising nanotheranostics platforms.

Several nanoconjugates have been proposed, varying in material, size and shape; some bringing current therapeutic approaches to the nanometer scale while others enact disruptive properties only possible by combination of different molecules and chemistries at the nanoscale (Conde et al.). For example, achieving controlled cellular responses of nanoparticles is critical for the successful development and translation of NP-based drug delivery systems. Conde et al. and Hong et al. (Pearson et al.) reported a complete survey on the most important factors for careful design of nanoparticles and the demand for precise control over the physicochemical and biological properties of NPs.

Liu et al. discuss the potential of star shaped nanoparticles in novel imaging approaches and strategies combining therapy and imaging in cancer. In fact, the potential of application of nanoconjugates in enhanced imaging strategies and platforms is discussed by Alcantara et al. with particular emphasis in current trends in molecular imaging for optimized management of breast cancer.

Theranostics of brain diseases such as brain cancer, is a daunting challenge due to the unique environment of central nervous system (Bhaskar et al., 2010). Yet passing the blood-brain barrier (BBB) is particularly difficult. The proper design of such engineered “nanocarriers” becomes very important in translocating the impermeable membranes of the brain to facilitate drug delivery. At the same time, it is also required to retain the drug stability and ensure that early degradation of drugs from the nanocarriers does not take place. In fact, Mahmoudi and Hadjipanayis reported a great opinion piece about the application of magnetic nanoparticles (MNPS) for the treatment of brain tumors and how MNPs will likely assume a larger role in brain cancer treatment in combination with other adjuvant therapies.

Talking about other adjuvant therapies, radiation and gene therapy have also gained momentum in the last years when using nanomaterials for cancer therapy. Cooper et al. reported how radiation therapy is one of the most commonly used treatments for cancer and which directions to follow for the future based on current state of nanoparticle-assisted radiation therapy.

Regarding gene therapy, Moreno and Pego reported a critical overview of using therapeutic antisense oligonucleotides against cancer and how difficult has been to get to the clinic. This is in fact not only a problem with gene therapy but a universal issue as whilst many pre-clinical data has been generated, a lack of understanding still exists on how to efficiently tackle all the different challenges presented for cancer targeting in a clinical setting.

Perhaps another interesting avenue in cancer nanotheranostics is the interfering effect of the immune system in the efficacy of proposed platforms. In fact, a clear perspective on the interaction between immune response and immune modulators is still missing from the general picture of nanotheranostics, not only in what concerns the organisms response to the systemic delivery of nanoconjugates that may hamper efficacy, but also the use of the immune response and nanoconjugates interaction with immune system as means to achieve higher and more directed/targeted therapy to the cancer site. As such, the effect and response of diverse properties of nanodiagnostics platforms in the organisms have been discussed by Clift et al. where nanoconjugates are discussed in terms of the immune response triggered after systemic delivery; whereas Conniot et al. and Pearson et al. (Dawidczyk et al.) have demonstrated how nanotheranostics may use and profit from the specific and unspecific immune response to enhance efficacy. Actually, cancer immunotherapy is nowadays consider a hot topic and a huge breakthrough in modern Science (Conde et al., 2015).

Gene expression has been targeted for silencing to avoid mutated protein function to exert its role in tumor progression. Nanotechnology based systems shows great promise in addressing novel genomic biomarkers that signal cancer cells, and do it with increased sensitivity that allow early detection of genome/genetic modifications that at the origin of cancer. Emergent technologies have been combined with nanoscale structures for directing to the site of interest with decreased side effects. The experience gathered thus far has shown that the next step in the effective translation of nanotheranostics into the clinics relates to the body's response to the nanoconjugates. What are the toxicity impacts of these devices and platforms? Are there enough data for the full chronic toxicity evaluation of the application of these systems? Is the immune system a friend or foe for nanotheranostics?

## Author contributions

All the authors contributed to this Editorial piece. All the authors read and revised the manuscript.

### Conflict of interest statement

The authors declare that the research was conducted in the absence of any commercial or financial relationships that could be construed as a potential conflict of interest.
